# Controversies in thyroid disease management in pregnancy

**DOI:** 10.1016/j.clinme.2025.100287

**Published:** 2025-01-17

**Authors:** Pushpa Singh, Kristien Boelaert

**Affiliations:** a University Hospital Birmingham NHS Foundation Trust, Diabetes Centre, Nuffield House, Mindelsohn Way, Edgbaston, Birmingham B15 2GW, UK; b Department of Applied Health, School of Health Sciences, Murray Learning Centre, College of Medicine and Health, University of Birmingham, Birmingham B15 2FG, UK

**Keywords:** Hypothyroidism, Hyperthyroidism, Pregnancy, Fertility, Thyroid autoimmunity

## Abstract

Adequate control of thyroid function is crucial for optimal pregnancy outcomes and neurodevelopment of the offspring, and testing for thyroid function is ideally performed using manufacturer- and gestation-specific reference ranges. While universal screening for thyroid dysfunction is not recommended, targeted case finding of women at risk of thyroid disease during pregnancy is advised. A number of controversies continue to fuel debate including: (i) the target range for thyroid stimulating hormone (TSH) in women with subfertility planning pregnancy, (ii) management of mild thyroid hypofunction before and during pregnancy, (iii) the treatment of thyroid peroxidase (TPO) antibody-positive euthyroid women with levothyroxine, (iv) the optimal choice of antithyroid treatment in women with hyperthyroidism. A significant body of evidence has accumulated in recent years, including large systematic reviews and meta-analyses of observational studies determining effects of thyroid dysfunction on pregnancy and fetal outcomes. In addition, outcomes from important randomised trials continue to inform current guidance on how to best care for women with thyroid dysfunction before and during pregnancy.

## Interpretation of thyroid function during pregnancy

Thyroid disease is common in women of childbearing age, and dynamic physiological changes to thyroid function need to be considered when interpreting test results. Thyroxine-binding globulin increases due to oestrogen stimulation, resulting in increased thyroxine (T4) and tri-iodothyronine (T3) production to maintain adequate concentrations of free thyroid hormones. High levels of human chorionic gonadotropin (hCG) have a thyroid-stimulatory effect and increase thyroid hormone production, resulting in decreased concentrations of thyroid-stimulating hormone (TSH). In addition, iodine requirements increase due to higher consumption for thyroid hormone production and increased renal clearance. The interpretation of thyroid function tests is further complicated by interference in the standard analogue immunological assays used to measure circulating thyroid hormones during pregnancy.^1^

Thyroid hormones are essential for maintenance of pregnancy, for placental function and for normal offspring development. While the fetal thyroid gland is formed and starts to function from 12–16 weeks’ gestation, the fetus is largely dependent on maternal thyroid hormone supply during the first 20 weeks and adequate neurodevelopment requires good control of maternal thyroid status throughout pregnancy.^1^ Current guidelines recommend the use of manufacturer- and gestation-specific reference ranges, which account for the physiological alterations, when interpreting thyroid function during pregnancy and to use an upper limit of 4 mIU/L for serum TSH if these are not available.^2^ Routine screening for thyroid dysfunction is not advised, but testing of subpopulations with specific risk factors, including women with subfertility and recurrent miscarriages, is recommended (Table 1).

**Table 1:** Subgroups of women in whom screening for thyroid dysfunction before and during pregnancy is appropriate.

A. Personal history of thyroid disease
• Previous thyroid surgery • Goitre or thyroid nodules • Previous history of thyroid dysfunction (overt or subclinical) • History of thyroiditis • TPO antibody positivity • Previous treatment with ^131^I • Ingestion of medications potentially affecting thyroid function (amiodarone, lithium, interferon, tyrosine kinase inhibitors) • Previous irradiation to the head or neck • Symptoms or signs indicative of thyroid dysfunction (weight loss, palpitation, signs of thyroid eye disease)
B. Associated autoimmune conditions
• Type 1 diabetes mellitus • Systemic lupus erythematosus • Sjogren’s syndrome • Antiphospholipid syndrome
C. History of subfertility
• Recurrent miscarriages • Stillbirth • Second-trimester miscarriage

## Prevalence of thyroid dysfunction before and during pregnancy

Overt hypothyroidism (TSH above and free T4 (fT4) below the normal refence range) occurs in 0.2% and subclinical hypothyroidism (TSH above and fT4 within the reference interval) in 2.4–6.0% of women seeking fertility, similar to prevalences in the general population. The prevalence of subclinical hypothyroidism increases substantially depending on the cut-off value for the upper limit of the TSH range and – if this is determined as 2.5 mIU/L – up to 20% of women may be diagnosed with the condition, potentially resulting in inappropriate levothyroxine replacement.^3^ Pregnancy and neonatal outcomes were not different in a large cohort of women in India started on levothyroxine for TSH concentrations above 2.5 mIU/L compared with those not treated.^4^ The most common underlying aetiology of hypothyroidism is thyroid autoimmunity and the prevalence of thyroid peroxidase antibody (TPOAb) positivity is 5–15% in women of childbearing age, with higher rates in those with recurrent miscarriages.^2^^,^^5^

Isolated hypothyroxinaemia is a pregnancy-specific condition of a low fT4 with normal TSH of unclear aetiology and occurs in 2.0–2.2% of pregnant women.^2^^,^^5^ Newly presenting hyperthyroidism in pregnancy is usually due to gestational transient thyrotoxicosis, which overall affects 1–11% of pregnant women with higher prevalences in those with hyperemesis gravidarum. Graves’ disease affects around 0.4% of women pre-conception and 0.2% during pregnancy.^6^

## Managing thyroid hypofunction in women with infertility

The definition of thyroid dysfunction in women with infertility or undergoing fertility treatment is similar to that in the general population and local general population reference ranges should be used. Measurement of serum TSH is recommended in women with infertility and recurrent miscarriage, since overt and subclinical hypothyroidism may contribute to the subfertility and since treatment with levothyroxine may improve fertility outcomes. Repeat thyroid function testing on a 3–6-monthly basis, in women known to have raised TPOAbs with TSH within the normal reference range, is advisable in view of the risk of progression to subclinical or overt hypothyroidism.^7^ In randomised controlled trials (RCTs), approximately 7–9% of euthyroid TPOAb-positive women developed (subclinical) hypothyroidism during a 12-month follow-up; cases were mostly detected pre-conception but also during pregnancy.^8^

For women taking levothyroxine replacement, a target TSH with the normal reference range but less than 2.5 mIU/L is advised. There is low-quality evidence that treatment of subclinical hypothyroidism in women seeking fertility with levothyroxine is associated with improved pregnancy and live-birth rates, but not for women with TSH between 2.5 mIU/L and the upper limit of the reference range, including those who are TPOAb positive.^7^ Significant physiological changes during assisted conception approaches may affect thyroid function^9^ and it is advisable not to check serum TSH within 2 weeks of an ovulation trigger, but ideally before ovarian stimulation or at 6 weeks of gestation.

## Managing hypothyroidism in pregnancy

Women of childbearing age taking levothyroxine for overt or subclinical hypothyroidism should be advised to empirically increase their dose by 25–30% as soon as they have a positive pregnancy test to meet the increased demands during pregnancy. This is most easily achieved by doubling the dose on 2 days of the week. Regular thyroid function testing should be performed on a 4-weekly basis until 20 weeks and subsequently once per trimester.^1^^,^^2^

The main risk factor for subclinical hypothyroidism during pregnancy is thyroid autoimmunity, and mild hypothyroidism is more prevalent in older women. The diagnosis is usually made incidentally in asymptomatic pregnant women presenting for routine obstetric care. Untreated subclinical hypothyroidism during pregnancy is associated with a higher risk of miscarriage, pre-eclampsia, placental abruption, preterm birth and small for gestational age. However, the absolute risk difference for these pregnancy complications as compared to euthyroidism ranges between +1 to +5%.^5^^,^^10^, ^11^, ^12^

Limited data from observational studies and one small RCT indicate lower rates of preterm birth and higher gross motor score in the offspring when treating women with serum TSH > 4.0 mIU/L with levothyroxine compared with observation or placebo in the first trimester.^13^ In two larger RCTs, the Controlled Antenatal Thyroid Screening (CATS) in the UK and Italy^14^ and the National Institutes of Health trial in the USA,^15^ levothyroxine started at a median gestation of 13 and 17 weeks respectively did not improve obstetric and neurodevelopmental offspring outcomes in women with subclinical hypothyroidism or isolated hypothyroxinaemia. Arguably, levothyroxine was started too late in the latter two studies and should ideally be given as soon as possible in the first trimester.

Recent evidence indicates potential harm with excessive doses of levothyroxine, including development of ADHD and autism,^16^ as well as potential adverse pregnancy outcomes including gestational diabetes mellitus, pre-eclampsia and preterm delivery, especially when treating women with TSH between 2.5 and 4.0 mIU/L with excessive dose of levothyroxine.^17^ A shared decision-making process discussing risks and benefits should be undertaken between the patient and physician.

## Thyroid autoimmunity in pregnancy

Risk factors for TPOAb positivity include other autoimmune disease, a first-degree relative with thyroid autoimmunity, greater age, obesity and nulliparity, while smoking has a protective effect and ethnic differences exist.^3^ TPOAbs reduce the functional capacity of the thyroid gland and are the most important risk factor for development of hypothyroidism. Pregnant women with euthyroid TPOAb positivity have higher risks of miscarriage and preterm birth, with absolute risk differences as compared to TPOAb-negative women ranging from +2% to +8% +2 to +3% respectively.^5^

Three randomised trials have shown that treatment of euthyroid TPOAb positivity with levothyroxine therapy started either pre-conception or in early pregnancy does not improve obstetric or newborn outcomes (Table 2).^18^, ^19^, ^20^ Importantly, there were no factors that modified the response to levothyroxine treatment, including TSH > 2.5 mU/L, previous miscarriage(s), maternal age or the TPOAb concentration. This suggests that the mechanism underlying the higher risk for TPOAb-positive women is not mediated through changes in thyroid hormone availability and that TPOAbs are a reflection of a more general susceptibility to autoimmunity, resulting in a higher risk of pregnancy complications.

**Table 2:** Summary of the most important RCTs evaluating effects of levothyroxine replacement in women who are euthyroid with raised TPO antibodies before and during pregnancy.

Trial	Country	Population	Intervention	Control	Primary outcome	Events intervention group	Events control group	Results
Wang 2017^19^	China	N=600 women (N=300 intervention and N=300 control) undergoing IVF-ET. TPO raised and biochemically euthyroid	Levothyroxine to titrate TSH: 0.1–2.5 mIU/L in first trimester 0.2–3.0 mIU/L in second trimester 0.3–3.0mIU/L in third trimester	No treatment	Miscarriage before 28 weeks’ gestation in women becoming pregnant	10.3%	10.6%	*Absolute RD =* -0.34% *95% CI=* -8.65– 8.12%
Dhillon-Smith 2019^18^	UK	N=940 women with history of miscarriage or infertility (N=476 intervention and N=476 control). TPO raised and biochemically euthyroid	Levothyroxine 50 µg OD	Placebo	Live birth after 34 weeks’ gestation	37.4%	37.9%	*P value=*0.74 *RR*=0.97 *95% CI=* 0.83–1.14 *Absolute RD=* -0.4% [-6.6–5.8]
Van Dijk 2023^17^	The Netherlands, Belgium, Denmark	N=187 women with two or more pregnancy losses (N=94 intervention and N=93 control). TPO raised and biochemically euthyroid	Levothyroxine 0.5–1.0 µg/kg depending on pre-conception TSH	Placebo	Live birth after 24 weeks’ gestation	50%	48%	*RR=*1.03 *95% CI=* 0.77–1.38

RD, Rate difference, RR, Relative risk, CI, Confidence interval.

## Hyperthyroidism in pregnancy

Suppressed serum TSH concentrations are common in pregnancy, especially in the first trimester as a consequence of high levels of hCG. It is important to distinguish gestational transient thyrotoxicosis (GTT) from Graves’ disease, since the former is a self-limiting condition which does not require antithyroid treatment and is not associated with adverse pregnancy outcomes.^21^ Prompt treatment of Graves’ disease is needed in pregnancy to reduce the risks associated with prolonged thyrotoxicosis including pregnancy loss, pregnancy-induced hypertension, intrauterine growth restriction, stillbirth and seizures disorder in offspring, as well as thyroid storm and congestive heart failure in the mother. GTT is more common in women with hyperemesis gravidarum and multiple pregnancies.^2^^,^^5^^,^^6^ A number of clinical and laboratory parameters help distinguish between the two conditions, as outlined in Table 3.

**Table 3:** Features distinguishing gestational transient thyrotoxicosis from Graves’ disease in patients presenting with thyrotoxicosis in the first trimester of pregnancy.

Feature	GTT	Graves’ disease
Symptoms of thyrotoxicosis BEFORE pregnancy	No	Often
Symptoms of hyperemesis gravidarum (nausea/vomiting)	Often	Usually not present
Personal of family history of thyroid disease	Usually absent	Often present
Presence of goitre	No/small	Diffuse goitre may be present
Signs of thyroid eye disease	No	May be present
fT3 concentration	Usually normal or mildly raised	Raised
TRAb concentration	Normal	Raised

Managing Graves’ disease pre-conception poses unique challenges. The desire for pregnancy should be discussed with all women of childbearing age. Treatment options including antithyroid drugs, radioactive iodine and surgery should be considered in a shared-decision making process.^2^ Women should be advised not to become pregnant while taking carbimazole and be changed to propylthiouracil (PTU), which is associated with lower teratogenic risks.^22^ Women opting for radioiodine pre-conception should be counselled regarding the risk of rise in TSH-receptor antibodies (TRAb) following radioiodine,^23^ which may cause fetal thyrotoxicosis.^24^ Ideally, conception should be avoided for 6–12 months or even longer following administration of radioiodine.

When antithyroid drugs are used in pregnancy, it is advisable to use the lowest possible dose and to discontinue antithyroid drugs whenever possible, especially since Graves’ disease usually improves in pregnancy.^2^^,^^22^ Regular thyroid function testing on a 2–4-weekly basis in women taking antithyroid drugs and measurement of TRAb are cornerstones to help optimise thyroid function control. If antithyroid drugs are still needed after 16 weeks’ gestation, PTU may be switched to carbimazole, since the period of organogenesis is complete and in view of potential hepatotoxicity associated with PTU.^2^ TRAb should be measured at 12 weeks’ gestation in any woman with a history of Graves’ disease, regardless of the treatment she has undergone, and generally levels of three times or more the upper limit of normal are associated with increased risks of fetal Graves’ disease. Repeat TRAb levels at 20 and 28 weeks’ gestation in women treated actively with antithyroid drugs at those time points or those with raised TRAb at 12 weeks. These measurements will inform the need for regular growth and heart rate monitoring in the fetus and predict the risk of neonatal thyrotoxicosis.^2^

## Conclusions

A significant body of recent evidence is changing the diagnosis and management of thyroid dysfunction in fertility and pregnancy. Thyroid function should be interpreted using assay- and pregnancy-specific reference ranges and levothyroxine is not recommended when TSH is within the reference range, even in the presence of thyroid autoimmunity. Hyperthyroidism should be carefully managed with regular thyroid function testing and measurement of TRAb. Individualised and shared-decision making is crucial, especially when treating mild thyroid hypofunction and when choosing preferred antithyroid treatments.

## Key points

• The presence of thyroid dysfunction during pregnancy should be determined using assay- and gestation-specific reference ranges, taking physiological and analytical changes into account.
• Subclinical hypothyroidism is associated with adverse pregnancy and neurodevelopmental consequences, but evidence that treatment with levothyroxine improves these outcomes is scant.
• There is no convincing evidence that treatment of TPO antibody-positive euthyroid women with levothyroxine improves fertility and pregnancy outcomes.
• Propylthiouracil is the preferred antithyroid treatment for women with hyperthyroidism before and in the first trimester of pregnancy.
• Appropriate counselling and shared decision making are crucial in managing women of childbearing age with thyroid dysfunction.

## CRediT authorship contribution statement

**Pushpa Singh:** Writing – original draft, Validation, Investigation, Conceptualization. **Kristien Boelaert:** Writing – review & editing, Validation, Investigation, Conceptualization.

## Declaration of competing interest

The authors declare that they have no known competing financial interests or personal relationships that could have appeared to influence the work reported in this paper.
